# Medicinal plants in the southern region of the State of Nuevo León, México

**DOI:** 10.1186/1746-4269-8-45

**Published:** 2012-12-11

**Authors:** Eduardo Estrada-Castillón, Brianda Elizabeth Soto-Mata, Miriam Garza-López, José Ángel Villarreal-Quintanilla, Javier Jiménez-Pérez, Marisela Pando-Moreno, Jaime Sánchez-Salas, Laura Scott-Morales, Mauricio Cotera-Correa

**Affiliations:** 1 Facultad de Ciencias Forestales, Universidad Autónoma de Nuevo León, Km 145 Carretera Nacional Linares-Cd. Victoria, A.P. 41, Linares, 67700, Nuevo León, Mexico; 2 Departamento de Botánica, Universidad Autónoma Agraria Antonio Narro, Buenavista, Saltillo, Coahuila, Mexico

**Keywords:** Ethnobotany, Medicinal plants, Uses, Nuevo León, México

## Abstract

**Background:**

Although the flora of the State of Nuevo León is well known, there are few records of ethnobotancial information. An ethnobotanical study was undertaken in order to know the medicinal plants used by people living at the scrublands and oak-pine forest areas in the southern Nuevo León. Collection of plants specimens and interviews were carried out among the people of the municipalities of Aramberri, Galeana, and Zaragoza. Since former studies in the region are scarce, the aim of this work was to record the medicinal species and their uses in the scrublands and oak-pine forest areas, of southern Nuevo León, Mexico, and also to know if there are differences in the number of species and number of uses knowledge by people.

**Methods:**

Field work was carried out over a 2 years period; useful plants were collected and a total of 105 people from 46 different villages were interviewed. A database was compiled using data collected by means of semi structured interviews. The data were analyzed by means of non-parametric statistics, using goodness-of-fit test (Chi-squared) (number of species known by people of each municipality, number of uses known by people of each municipality), Chi-squared modified to incorporate the Yates Correction (number of species known by people living at scrublands and oak-pine forest); the Kruskall-Wallis test (number of species known by women and men of the three municipalities), and the Spearman’s rank correlation coefficient (age and number of species known, and age and number of uses).

**Results:**

A total of 163 medicinal plant species were recorded in the study area, comprising 108 wild and 55 cultivated plants. A total of 117 species were recorded in the oak-pine forest, and 111 in the scrublands area, a total of 68 were recorded in both areas; 68 medicinal species are used in all three municipalities, 40 wild and 28 cultivated. We documented 235 different medicinal uses. The most common plant parts used for medicinal purposes were found to be leaves (123 species), stems (55), fruits (28), roots (17), and bark (14). No differences were noted in the number of medicinal plant species identified among people, but differences were significant in their knowledge with respect to the number of uses among people of the three municipalities studied; people from both, scrublands and oak-pine forest know similar number of species and number of uses. Men and women of the three different municipalities knew statistically the same number of species and number of uses. There was no correlation between resident’s age and number of species known and resident’s age and number of uses either in Galeana or in Aramberri, but, there was high correlation among these variables in Zaragoza.

**Conclusion:**

In southern Nuevo León people use at least 5% of the total State flora as medicinal plants, and most of these species are included in few plant families. Most of medicinal species are wild and indigenous to the region. The two most important major plant communities, scrublands and oak-pine forest provide almost the same number of medicinal species. A third of the medicinal flora recorded are used in all three municipalities, most of them are wild. Leaves, stems and fruits are the plant parts most commonly used for healing, and boiling is the most common method used for this purpose. Men and women from the three municipalities are familiar with nearly the same number of species; however, their knowledge of the number of uses varies significantly. In Galeana and Aramberri there was no correlation between a person’s age and number of species recognized, however, in Zaragoza, there existed a high correlation between these two factors.

## Background

Mexico is one of the countries with the highest diversity of plants in the world [1-3]. Currently, the total number of plant species is estimated at almost 22,800 species [4], where oak-pine forest has nearly one quarter of the flora, while xeric scrublands and grasslands constitutes almost 20% [5]. Mexico has the second highest number of medicinal plants species with almost 4,500 recorded after China (almost 5,000) [6]. Medicinal plants are still the most commonly known and accessible resource for poor people in Mexico [7], and, there are recorded studies for almost 5,000 to 7,000 beneficial species in Mexico [8]. Most studies concerning ethnoecology [9] or about medicinal plants in Mexico have been carried out at south of the Tropic of Cancer, in the calid-humid region, especially those works concerning general ethnobotany [10-13], or regional ethnobotany pertaining to one or two species [14-22], and also systematic-ethnobotany studies [23], as well as ethnobotany and phytochemical compounds [24,25].

In the north of Mexico most of ethnobotanic studies have been carried out in the northwestern States [26-30], and only a few studies have been carried out from the northeastern area [31].

In spite of the rich plant diversity, with almost 3,175 species of vascular plants found in the State of Nuevo León [32] and its heterogeneous plant associations, constituting 13 main plant communities, almost nothing is known about its useful plants, except for the study of the Cumbres de Monterrey National Park in the central region of the State [33].

The southern region of the State of Nuevo León comprises five municipalities, Aramberri, Doctor Arroyo, Galeana, General Zaragoza and Mier y Noriega. The study was carried out in three of them: Aramberri (2,809 km^2^), Galeana (6,740 km^2^) and General Zaragoza (1,289 km^2^), all of them include the scrublands and oak-pine forest as their main plant communities in their geopolitical borders [34], all of them have basic health services. The economy in this region is based mainly on agriculture (corn, oat, wheat, potatoes, peas, pepper, garlic, fig, avocado, nuts, onion, beans and alfalfa), fruit production (apples, plums, apricots, nuts, and peaches, mostly in cool climates), textile and wood industry as well as the sale of cattle. In the southern area of Nuevo León women are responsible for domestic activities such as child care while men pursue different economic activities. Women participate actively in the home economy by selling products fabricated at home such as candies, aguamiel (mead), maguey syrup, tortillas, as well as resources obtained from countryside such as pine (*Pinus* spp.) or oak (*Quercus*) wood, fruits called chochas or flor de palma (*Yucca carnerosana* flowers) and dried medicinal. This last activity implies long hikes through canyons, mountains and valley to gather them, loading the product on donkey, mule or horse. People of southern Nuevo León use plants by empirical knowledge in different ways, such as cure of diseases, fuel, food, fibers, wood, among others. However, the medicinal use of plants are still an alternative against health problems, especially in old people with praxis who live in villages far away from the largest cities where health care institutions are found. People living in the villages studied would dry medicinal plants in order to store them in paper or plastic bags hung in bundles from the ceiling, and then use them daily as medicine, condiment, tea, such as *Litsea pringlei, Matricaria recutita, Trixis californica* var. *californica*, *Larrea tridentata, Flourensia cernua, Poliomintha longiflora, Lippia graveolens,* and *Equisetum laevigatum* (information recorded in the ethnobiological research, according to local people).

The focus of this study is to augmenting the documented knowledge of medicinal plants from the two major plant communities, the scrublands and the oak-pine forest areas of the southern part of the State of Nuevo León, México.

We wanted to know if 1) there are differences in the number of plant species known and the way in which each is used (number of uses) by people of the three different municipalities, our hypotheses is that there are not significant differences in either the number of plants for which local residents were cognizant nor number of uses. We also wanted to know, if 2) there are differences in the number of plant known and the number of uses people use in both areas, since a higher plant number was recorded in the oak-forest area than in the scrublands. Our hypotheses is that people living in forest areas knows a greater number of plants and a wider number of uses than the ones living in the scrublands area. Most of men interviewed were farmers, cowboys or shepherds, and most of the women interviewed were housewives, 3) We wanted to address whether the men questioned knew a larger number of plant species and had a greater knowledge of the number of uses than women? Our hypotheses are that men in close contact with nature would know a larger number of plant species than women, but, women would know a greater number of uses for each plant species than men, since women interact daily with more people. Since the age is highly correlated with global knowledge, we wanted to test if 4) if older people know more number of species and more number of uses than younger people. Our hypotheses are that older people know more number of species and more number of uses than their younger counterparts.

## Methods

### Study area

**Figure 1:**
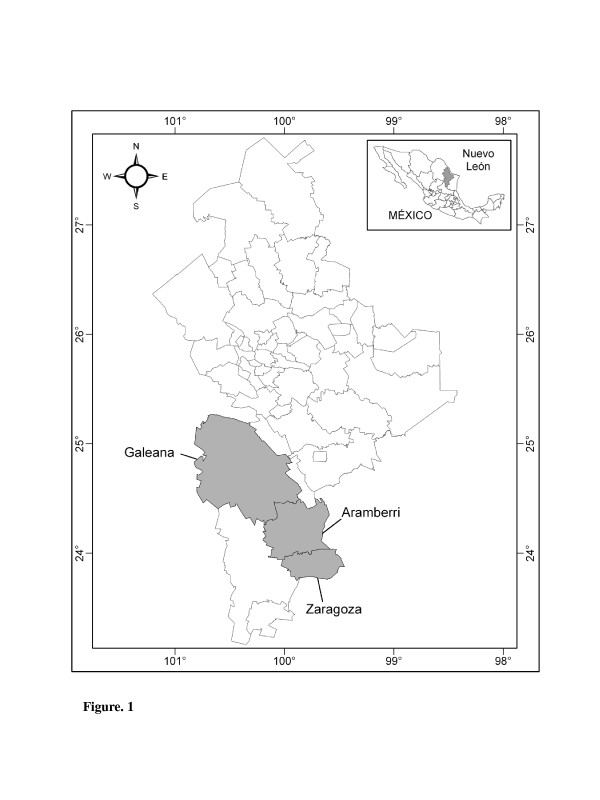
**Study area, the three municipalities studied, Aramberri, Galeana and Zaragoza** .

The three municipalities (Aramberri, Galeana, and Zaragoza) are located in the Sierra Madre Oriental physiographic province, and in two physiographic subprovinces of it, Gran Sierra Plegada and Sierras y Llanuras Occidentales (Figure 1), the Gran Sierra Plegada subprovince has temperate climate C(W) with summer rains, sheltering mainly oak, oak-pine, and pine forest, dominated by *Pinus pseudostrobus, P. teocote, P. greggii, P. cembroides, Abies vejari, Quercus polymorpha, Q. risophylla, Q. mexicana,* and *Q. hypoleucoides*. The altitudes ranging from 1,800 to 3,650 m, and the mean temperature ranges from 13°C to 17°C, while Sierras y Llanuras Occidentales subprovince has semi dry, semi warm climate C(E)(W), including summer rains, the vegetation is mainly composed by scrublands with heterogeneous physiognomy, dominated by *Larrea tridentata, Flourensia cernua, Acacia vernicosa, Opuntia* spp., *Yucca* spp., *Agave lechuguilla, A. striata, Nolina* spp., and *Parthenium* spp., the altitudes oscillates from 1,300-1,780 m with a mean temperature ranging from 20°C to 25°C [35].

### Field work

Information about number of species and number of uses and common names were obtained from June to December 2009 (30 interviews), March to December 2010 (60 interviews), and from March to June 2011 (15 interviews). Ethnobotanical information was collected through semi-structured interviews, and according to the efficiency decreasing law [36]. Three main questions were asked, 1) what it its medicinal use (stomach ache, diabetes, diarrhea, headache, inflammation, cough, kidney diseases, wounds, colic, etc.), 2) how is the plant prepared (boiled, raw, toasted, or macerated), and 3) what part of the plant is used (root, stems, leaves, inflorescences, buds, flowers, fruits, or seeds). We acknowledge methodological limitations such as the strong gender bias in the sample. Therefore, the interviews were stopped when information efficiency recorded was lower than 4% of the total species and 3% of the number of uses for both, women and men. To correlate the common name with its scientific name, we collected plant specimens in the field, showed them to those interviewed in order to confirm the correct name of each and inquired about the use of each species. In addition, we made field trips with the informants for *in situ* identification of plants and collected voucher specimens of all the species recorded in this study.

### Interviews

The age of the people interviewed ranged from 33 to 92 years old. Seven age ranges were selected [37] (Table 1). Sixty one percent of the those interviewed were older than 60 years, while 39.1% of them were younger than 60 years old; 54 of the interviews were carried out in the scrublands area while 46 were conducted in the oak-pine forest region; 73 interviews were made among women and 28 among men, because it was easier to locate women at home when the interviews were conducted (Table 1). All those people interviewed born or had been residents of this area for at least 30 years. All the interviews were performed in Spanish, since the informants do not speak another language and do not belong to another ethnic group. Notes were taken during all conversations. All information concerning medicinal plants was recorded, including plants names and their use. Voucher specimens of each plant species we collected was deposited together with all the relevant data in the herbarium (CFNL, Linares, N. L.).

**Table 1:** Age ranges, number of interviews by range, and number of men and women interviewed by municipality in the southern Nuevo León, México

**Range age**	**Number**	**Galeana**	**Aramberri**	**Zaragoza**
**(years old)**	**of interviews**	**(Women, Men)**	**(Women, Men)**	**(Women, Men)**
30-39	14	6 (5, 1)	4 (3, 1)	4 (3, 1)
40-49	9	3 (2, 1)	3 (2, 1)	3 (2, 1)
50-59	18	7 (6, 1)	6 (4, 2)	5 (3, 2)
60-69	33	12 (10, 2)	11 (9, 2)	10 (8, 2)
70-79	22	8 (5, 3)	7 (5, 2)	7 (5, 2)
80-89	7	3 (2, 1)	2 (3, 1)	2 (1, 1)
90>	2	1 (W)	1 (M)	0
TOTAL	105	40	34	31

*W* = Woman, *M* = Man.

### Data analysis

The Goodness-of-fit test (Chi squared test) was used for questions 1 and 2, since they are nominal variables; however, the Yates Correction was incorporated in question 2, a correction for continuity. The Kruskall-Wallis (*H*) test was applied to question 3 in order to test for differences among medians. In question 4, age, number of species and number of uses were transformed to ordinal variables and analyzed by the Spearman’s rank correlation coefficient (*r*_S_) [38].

## Results

### Diversity

The total medicinal flora recorded represent the 5.1% of the total flora of Nuevo León (Additional file 1: Appendix 1); six of the families includes 41.7% of the total medicinal species; four main growing forms were recorded; two-thirds of the species recorded were wild, and one-third were cultivated. Almost the same value, two-thirds and one-third were also for plant provenance respectively; scrublands and oak-pine forest shelter almost the same number of medicinal plants. A higher number of wild and cultivated medicinal plants were recorded at Galeana (Table 2). Sixty eight plants of medicinal species were used in the three municipalities, 40 wild and 28 cultivated. There was a concordance among the common names used by individuals for most of the plants in both subrpovinces and the three municipalities, predominantly because almost all plants have only one name, except for four. Arnica is applied to two species (*Grindelia inulioides* var. *inulioides* and *Trixis californica* var. *californica*), betónica (*Hedeoma palmeri* and *Monarda citriodora* var. *citriodora*), orégano (*Poliomintha longiflora* and *Lippia graveolens*), and cedro (*Juniperus deppeana* and *Cupressus arizonica*). According to their similar physiognomy and despite their high species diversity, all species belonging to the genus *Opuntia* mentioned have different common names.

**Table 2:** Total diversity, families with the highest number of medicinal species, growth forms, plant origin, plant procedence, species by major plant community, and number of species by municipality in the southern Nuevo León, México

Total flora recorded	58 families, 136 genera, and 163 species
Families with the highest number of medicinal species	Asteraceae (18), Lamiaceae (15), Agavaceae (8), Leguminosae (8), Cactaceae (8), Euphorbiaceae (6), and Apiaceae (5)
Main growth forms	Herbaceous (94) shrubs, (39), trees (23), succulent (7).
Origin	Wild (108), Cultivated (55)
Procedence	Autochthonous (117), introduced (46)
Species number by major plant community	Oak-pine forest (117), Scrublands (111)
Species recorded by municipality (c = cultivated, w = wild)	Galaena (c = 48, w = 83); Aramberri (c = 40, wild = 83), Zaragoza (c = 36, w = 60)

### Number of uses for genera and species

**Figure 2:**
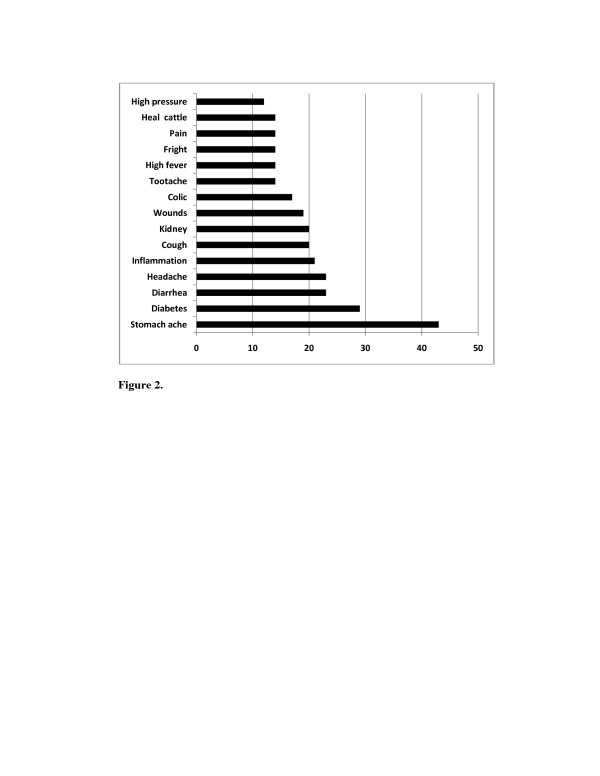
**Number of medicinal species mentioned to heal the most important ailments in the southern region of the State of Nuevo León, México** .

Sixteen genera include at least two species used for medicinal purposes, and 16 species include at least nine different medicinal uses (Table 3). We recorded 235 different medicinal uses in this region. Several of the species recorded were used to alleviate, control or to heal different diseases, among the most common diseases healed by these plants are stomachache, diabetes, diarrhea, headache, and inflammation (Figure 2).

**Table 3:** Genera with the highest number of medicinal species, and species with the highest number of medicinal uses in the southern Nuevo León, México

*Prunus*	3	*Ruta graveolens*	16
*Mentha*	3	*Malva parviflora*	14
*Yucca*	2	*Marrubium vulgare*	14
*Tagetes*	2	*Mentha piperita*	13
*Buddleja*	2	*Trixis californica* var. *californica*	13
*Cylindropuntia*	2	*Matricaria recutita*	13
*Opuntia*	2	*Tagetes lucida*	11
*Pinus*	2	*Larrea tridentata*	10
*Chenopodium*	2	*Rosmarinus officinalis*	10
*Carya*	2	*Artemisia ludoviciana*	10
*Hedeoma*	2	*Pelargonium hortorum*	10
*Litsea*	2	*Buddleja cordata* ssp. *tomentella*	9
*Acacia*	2	*Ocimum basilicum*	9
*Eysenhardtia*	2	*Chrysactinia mexicana*	9
*Phaseolus*	2	*Schinus molle*	9

### Usage

**Figure 3:**
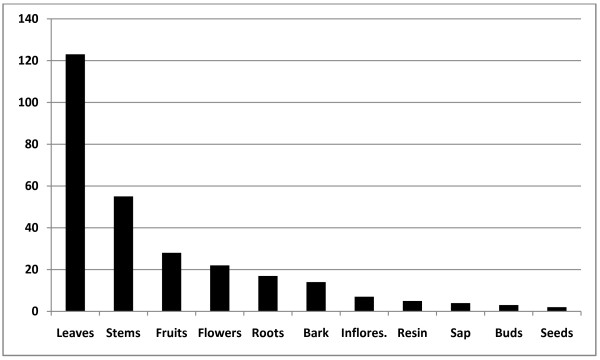
**Part of the plant more commonly used for medicinal purposes in the southern region of the State of Nuevo León, México** .

The most common plant parts used for medicinal purposes were leaves, stems, and fruits (Figure 3). Of the total flora, four main preparation methods were recorded: *a*) boiled, *b*) raw, *c*) toasted, and *d*) macerated. Of the four, boiled one stands out from the others by far. Leaves from 104 different plant species were used for different remedies by boiling, followed by stems (44), flowers (21), bark (11), inflorescences (6), and resin (5). In an effort to heal some diseases, the whole plant, parts of them, and even the sap of some plants were used raw, without any prior preparation, and, in this case, also, the leaves were also the plant part most commonly used (39 different species), followed by stems (20), bark (7), sap (7), buds (1), and seeds (1). Occasionally, plant parts should be toasted before using them in healing; we recorded 10 different species in which toasted leaves are used for different remedies. Also, other plant parts used in this way include, stems (6 species), inflorescences (2), flowers (2), buds (1), and bark (1). Uncommon, but also utilized were, macerated parts of a few species such as *Agave lechuguilla* (root)*, Aloe vera* (leaves)*, Opuntia engelmanii* (stems)*, Opuntia ficus-indica* (stems and root)*, Chenopodium ambrosioides* (stems and root)*, Allium sativum* (stems and bulbs), and *Solanum tuberosum* (roots).

### Knowledge about plant species and their uses

In order to ascertain the knowledge informants had about the number of species and the number of uses, people from the municipalities in question were interviewed, we recorded 130 different plant species in Galeana, 123 in Aramberri, and 99 in Zaragoza, using the Chi squared test did not show significant values among the variables (Table 4). Concerning to the number of uses in which each plant is used in each municipality, people form Galeana mentioned 173 different uses, Aramberri 156, and Zaragoza 113, the Chi squared test showed significant values about number of uses (Table 4). In relation to the different number of species and their number of uses, individuals living in the scrublands and oak-pine forest areas, the Chi squared test did not yield significant values for the number of species nor for their different number of uses (Table 5). A comparison of knowledge between men and women showed no significant differences in number of plants (Table 6) nor the varied number of uses (Table 6); as we stated before, we acknowledge the strong gender bias in the sample, but, according to the information recorded during the interviews, we stopped them when information efficiency recorded was lower than 4% of the total species and 3% of the number of uses for both, women and men, however, we also aknowledge that results about the number of species and the number of uses obtained for men should be treated with caution due to the small number of interviews with them. The Spearman’s rank correlation coefficient (*r*_S_) did not show significant values neither for the number of species and age nor for the number of uses and age in women (Table 7) and men (Table 8) from Galeana. The Spearman’s rank correlation coefficient (*r*_S_) did not show significant values neither for the number of species and age nor for the number of uses and age in women (Table 9) and men (Table 10) from Aramberri. In contrast, the *r*s values for number of species and the number of uses for both, women (Table 11) and men (Table 12) were statistically significant in Zaragoza.

**Table 4:** **Number of species and number of uses mentioned for people of the three municipalities and the *X***^***2***^**test**

	**Galeana**	**Aramberri**	**Zaragoza**	***X***^***2***^**(Yates correction incorporated)**
Number of species recorded	130	123	99	*X*^*2*^ = 5.11, *d.f.* = 2, α = 0.05
Number of uses recorded	173	156	113	*X*^*2*^ = 13, *d.f.* = 2, α = 0.05

**Table 5:** **Number of species and number of uses recorded for people living at the scrublands and oak-pine forest areas and the *X***^***2***^**test**

	**Scrublands area**	**Oak-pine forest area**	***X***^***2***^**(Yates correction incorporated)**
Number of species recorded	111	117	*X*^*2*^ = 0.011, *d.f.* = 1, α = 0.05
Number of uses recorded	210	214	*X*^*2*^ = 0.011, *d.f.* = 1, α = 0.05

**Table 6:** **Number of species and number of uses recorded for men and women of the three municipalities and the Kruskall-Wallis test (*H*)**

	**Galeana**	**Aramberri**	**Zaragoza**	**Test *H***
				**(Kruskall-Wallis Test)**
Number of species mentioned by men (Sum of ranks)	113.9	167	88.1	*H* = 1.75, k = 3, n = 9
Number of species mentioned by women (Sum of ranks)	199.66	136.66	124.66	*H* = 2.58, k = 3, n = 11
Number of uses mentioned by men (Sum of ranks)	123.66	149.33	100.99	*H* = 0.28, k = 3, n = 9
Number of uses mentioned by women (Sum of ranks)	200.5	143.5	113.99	*H* = 2.2, k = 3, n = 11

**Table 7:** **Age, number of species and number of uses mentioned by women of Galeana, Nuevo León and the Spearman’s rank correlation coefficient (*r***_***s***_**)**

**Age**	**Number of species**	**Number of uses**
35	29	32
35	10	9
36	20	22
39	12	14
45	27	39
54	38	77
54	48	51
55	47	56
59	19	26
60	23	25
63	17	26
66	36	38
67	9	9
68	18	19
68	28	34
69	10	17
74	25	33
80	29	15
80	15	42
88	13	16
	*r*_*s*_ *=* 0.19, α = 0.05	*r*_*s*_ *=* 0.0, α = 0.05

**Table 8:** **new. Age, number of species and number of uses mentioned by men of Galeana, Nuevo León and the Spearman’s rank correlation coefficient (*r***_***s***_**)**

**Age**	**Number of species**	**Number of uses**
33	12	12
60	18	27
62	5	5
63	38	40
66	5	6
67	57	53
72	8	8
89	12	11
92	17	18
	*r*_*s*_ *=* 0.05, α = 0.05	*r*_*s*_ *=* 0.0 α = 0.05

**Table 9:** **Age, number of species and number of uses mentioned by women of Aramberri, Nuevo León and the Spearman’s rank correlation coefficient (*r***_***s***_**)**

**Age**	**Number of species**	**Number of uses**
30	48	61
32	31	39
33	7	7
35	10	10
36	10	13
37	12	14
40	24	14
40	14	26
40	2	2
50	42	44
50	36	40
52	4	5
53	46	48
55	38	57
58	46	34
63	66	83
65	11	8
66	8	11
67	24	24
69	29	31
70	15	15
74	5	4
74	27	38
79	13	15
80	4	4
80	17	25
83	12	13
	*r*_*s*_ *=* -0.11, α = 0.05	*r*_*s*_ *=* 0.05, α = 0.05

**Table 10:** **Age, number of species and number of uses mentioned by men of Aramberri, Nuevo León and the Spearman’s rank correlation coefficient (*r***_***s***_**)**

**Age**	**Number of species**	**Number of uses**
45	4	5
48	20	23
55	54	95
55	54	67
60	13	16
64	33	38
72	31	34
74	33	39
77	4	4
90	19	23
	*r*_*s*_ *=* 0.09, α = 0.05	*r*_*s*_ *=* 0.1 α = 0.05

**Table 11:** **Age, number of species and number of uses mentioned by women of Zaragoza, Nuevo León and the Spearman’s rank correlation coefficient (*r***_***s***_**)**

**Age**	**Number of species**	**Number of uses**
46	20	23
57	18	20
58	17	19
60	11	11
63	19	23
63	12	13
63	16	23
65	3	6
65	6	9
65	13	13
67	9	12
74	19	22
76	23	30
	*r*_*s*_ *=* 0.87, α = 0.05	*r*_*s*_ *=* 0.88, α = 0.05

**Table 12:** **Age, number of species and number of uses mentioned by men of Zaragoza, Nuevo León and the Spearman’s rank correlation coefficient (*r***_***s***_**)**

**Age**	**Number of species**	**Number of uses**
50	17	20
60	13	17
69	16	17
70	5	6
70	5	5
75	5	6
78	15	14
78	21	32
78	5	5
83	8	9
	*r*_*s*_ *=* 0.92, α = 0.05	*r*_*s*_ *=* 0.94 α = 0.05

### Economic importance of some wild and cultivated medicinal plants in the study area

Besides their medicinal use, several of the plant species recorded play an important role in economic activities of these small cities as well as in communes (ejidos), and villages. Some wild medicinal plants such as *Castela erecta* var. *texana, Equisetum laevigatum, Chenopodium ambrosioides, Artemisia ludoviciana, Larrea tridentata, Gnaphalium canescens*, and *Artemisia leudoviciana* are commonly collected in the country side, around the towns. After they are collected, plants are commonly dried in shaded places for several days or a few weeks and separated into parts according to their use. Dried plants are sold in tied bundles in town or elsewhere. Several species of trees are widely used as fleshy fruit producers such as *Persea americana* var. *drymifolia* (avocado)*, Prunus domestica* (plum)*, Prunus persica* (peach)*, Punica granatum* (grenade)*,* or dry fruit producers such as *Carya illinoiensis* (hickory). The most important ones by far are avocado and hickory, since there are extensively used crops. Both products are in high demand throughout Mexico. Several species of *Quercus* are used to produce good quality (durable) charcoal*,* and the hardwood of *Helietta parvifolia* makes it one of the most frequently used for the construction of wooden houses, roofs, and fence posts. Alfalfa (*Medicago sativa*) is cultivated and used for fodder production, and it is sold in bales in the area. A seasonal product is the chile piquín (chilli pepper) (*Capsicum annum* var. *glabriusculum*). It is collected mainly in the second half of the year, and sold in almost all markets in the area, reaching at times (when the harvest is poor) to $18.00 dollars per kg. A common product sold in many houses in the three municipalities is “aguamiel”, the sap is commonly extracted from agave stems, rather than the leaves. A common species used for this purpose is *Agave macroculmis* (maguey)*,* since its large stems dimension, produces a generous amount of this product. A half gallon bottle mead-filled is sold for almost $5.00 dollars. Each plant of maguey (according to comments of individual interviewed) produces 2-3 liters of mead every 36-48 hours, depending on the season; maguey is more productive on hot seasons. Other product obtained from the maguey is the “quiote”. During holy week (April), a hole is dug on the ground and the scape (big peduncle of the inflorescence) is excised from the plant. Then the scape is introduced into the hole and cooked. The cooking time for the scape is one day. After that, the scape is sliced in small parts called quiotes. Berros (watercress) (*Rorippa nasturtium-aquaticum*) grows abundantly in shallow streams. The plant is collected and sold in markets to make salads together with *Opuntia ficus-indica*, another plant cultivated in many gardens of this area. Cladodes are chopped and sold in plastic bags. A European weed [39], *Eruca sativa* called regionally as colesia*,* invades huge abandoned cultivated surfaces in southern Nuevo León, however, people from this area agree that this plant is a good forage for their cattle. People gather several tons of this plant after rainy season, and feed the livestock with it, fresh or dried; this plant is also sold in bales. There are two wild species with high economic value for the residents, *Poliomintha longiflora* (located in oak-pine forest area) and *Lippia graveolens* (scrublands)*,* both are known as orégano and used mainly as condiment. Gathered plants are dried in shaded areas, the dried parts are sold in small (100 gr) or medium (250 gr) plastic bags, and the price reaches $7.00 to $12.00 dollars per bag, respectively. Another plant sold in north of Mexico is *Turnera diffusa* (yerba del vanado or damiana), an aphrodisiac (ordinary people call it poor man’s viagra). The properties attributed to this species, make it a high demand product. It is also sold in tied bundles or in dried small parts in plastic bags. In regional markets it is also common to find the laurel (*Litsea pringlei*). Not as common as the aforementioned, but its dry leaves are commonly used to cook and spice up the food. This plant also packaged in small plastic bags or in tied bundles. *Agave lechuguilla* (lechuguilla) produces fibers which are used to manufacture cords and bags. However, the sparse return from this activity makes it economically unfeasible. Even so, a few residents continue to pursue it by selling it to cord producer as a mean of subsistence. One kilogram of *Agave lechuguilla* fiber is currently sold for almost $3.00 dollars (information recorded in the ethnobiological research, according to local people).

## Discussion

### Diversity

A few plant families (10.34%) make up almost the half of the medicinal species. Most of the medicinal species are herbaceous and shrubs in southern Nuevo León. Most of the species used as medicinal plants are wild and native. Indigenous scrublands and oak-pine forest areas provide almost the same number of medicinal species for local people, while 40% of them are commonly used in all the three municipalities. Almost all (but *Mentha*) genera have two or more medicinal species and are native plants. On the other hand, half of the medicinal species comprising the highest number of uses are introduced and cultivated. People in southern Nuevo León use all plant parts, but boiled leaves and stems are the most common plant parts used for medicinal purposes. Almost all medicinal species with fleshy stems (Cactaceae) and fleshy roots (potatoes) are used raw and macerated.

### Knowledge on species and their uses

People interviewed in the three municipalities have knowledge of nearly the same number of species, averaging 24, 23 and 21, but different number of uses, 29, 25, and 16 for Galeana, Aramberri, and Zaragoza respectively. Most of medicinal plants recorded are well known by people of the area, however, there is a significant difference in the number of uses. People from Galeana and Aramberri, know more number of uses and number of species than those from Zaragoza. Those 60 years old or younger from Zaragoza know less number of uses of plants than the ones from Galeana and Aramberri. Nevertheless people living in scrublands or oak-pine forest areas, know the same number of species and number of uses, they mentioned plants and their use distributed in the two main plant communities. Both, women and men of all the three municipalities know statistically the same number of plant species and their number of uses, however, the results obtained for men should be treated with caution due to the small number of interviews with them. Men and women of different ages interviewed in Galeana and Aramberri know the same number of plant number of uses, but, in Zaragoza, older people know more number of species and number of uses than younger people. The men and women from Zaragoza 60 years old and up have average on knowledge of 24 species and 26 uses, while in Galeana and Aramberri averaged 19 and 21 respectively.

### Similarities between ethnomedical studies in northeastern Mexico and Mesoamérica

The length and breadth of Mexico show strong similarities regarding wild and cultivated medicinal taxa and their uses. From Veracruz the plant families Asteraceae, Piperaceae, Leguminosae and Euphorbiaceae agglutinate the most important genera and species with medicinal use [40]. In arid and semiarid lands in the south of Puebla, 98 medicinal species were recorded [41], of those, 18 cultivated (*Carica*, *Citrus*, *Coriandrium*, *Cucurbita*, *Ficus*, *Lycopersicon, Persea*, *Petroselinum*, *Phaseolus*, *Prunus*, *Psidium*, *Sechium*, and *Zea*), and 4 wild species (*Amaranthus*, *Castela*, *Opuntia*, and *Quercus*) were found also to be common in our area. Furthermore, leaves are the most common parts used in the healing process; of the 46 medicinal species recorded in the Tehuacán-Cuicatlán Valley [42], 12 wild and 16 cultivated are also found in our study area, cooking being the most common method; cultivated medicinal plants in north of México and Mesoamérica are used as fruit trees, among those are *Prunus domestica*, *P. persica*, *P. serotina*, and *Ficus carica*[43]; some medicinal species recorded form Zitácuaro, Michoacán [44] are used in similar way in southern Nuevo León such as, *Chenopodium ambrosioides*, *Lycopersicon esculentum*, *Malva parviflora*, *Opuntia ficus-indica*, *Portulaca oleracea*, *Punica granatum*, and *Tagetes lucida*; a large number of species and number of uses are shared between Ocotlán, Oaxaca and our study area, 83 genera and 32 medicinal species recorded in Ocotlán [45] are also found in southern Nuevo Leon, and the leaves, stems, flowers, sap, roots, and fruits are the most common plant parts used. Some of these important medicinal species belong to the genera *Aloe*, *Apium*, *Bouganvillea*, *Caesalpinia*, *Carya*, *Citrus*, *Commelina*, *Lippia*, *Ocimum*, *Persea*, *Psdium*, *Punica*, *Rosmarinus*, *Ruta*, *Sambucus*, *Sechium*, *Solanum*, *Tecoma*, and *Tanacetum*. Gastrointestinal and skin disorders are a common problem in children in Mexico, and the group of medicinal plant species used by the Zapotec, Maya and Nahuas ethnias to heal them are *Chenopodium ambrosioides*, *Psidium guajava*, *Artemisia ludoviciana*, *Ruta chalepensis*, *Citrus limon*, *Mentha piperita*, *Matricaria recutita*, *Marrubium vulgare*, *Punica granatum*, *Turnera diffusa*, *Anoda cristata*, *Eucalyptus camaldulensis*, *Bouganvillea glabra*, and *Citrus limon*, [46]. Some of these plants are used to heal the same disorders in southern Nuevo León. Thirty four Cucurbitaceae species in Mexico have medicinal properties, and in most of cases, almost all of their parts are used for this purpose [47]. Some of these genera such as *Cucumis*, *Cucurbita*, *Cyclanthera*, *Sechium*, *Apodanthera* thrieve in Nuevo León, and are used in the same way. Almost 50 species of *Quercus* are used as medicinal plants to heal tooth problems, gastritis, diarrhea, burns, vaginal infections, heart, kidney diseases, cough, nervousness, muscle pains and diabetes [48]. Some of these uses are also common in southern Nuevo León. Of the 32 species of useful *Mimosa* species recorded for Mexico [49], one of them, *Mimosa malacophylla* is also indigenous to our study area and likewise used in a similar way. In Querétaro, several species of *Agave* provide maguey sap (mead) to heal some diseases and also to produce a fermented beverage called pulque [50,51]. In southern Nuevo León, mead and pulque are medicinal and economically relevant medical product used to combat tuberculosis (*Mycobacterium tuberculosis*) in cattle, it is obtained from *Agave lechuguilla*[52]. Resin from some *Pinus* species is used as ointment on wounds, bone fractures, backaches and wounds of domestic animals [53]. In southern Nuevo León *Pinus pseudostrobus* is used in the same way. Among the most common species used to control o heal diabetes mellitus in Morelos, are *Acacia farnesiana*, *Allium sativum*, *Artemisia ludoviciana*, *Baccharis salicifolia*, *Castela erecta* var. *texana*, *Citrus sinensis*, *Marrubium vulgare*, *Medicago sativa*, *Ocimum basilicum*, *Olea europaea*, *Opuntia imbricata*, *Pathernium hysterophorus*, *Phalaris canariensis*, *Physalis philadelphica*, *Punica granatum*, *Tagates erecta*, *Taraxacum officinale*, *Tecoma stans*, and *Zea mays*[54]. Some of these species are also used in the same way in our region; several species such as *Lippia graveolens*, *Prosopis leavigata* and *Urtica dioica* are used by residents to heal different infectious diseases in Huautla, Morelos [55], the authors state the importance of ethnopharmacology as a guide in the selection of plants for the discovery of bioactive compounds. These plants studied among others, also distribute in southern Nuevo León and are also used to heal several types of diseases. *Lophophora williamsi* (peyote)*,* and *Cannabis sativa* (marihuana) are a singular species due to its chemical contents of mescalina, with psicoactivity and potency similar to the salvinorina found on *Salvia divinorum*[56], and the tetrahydrocannabinol compounds respectively, in southern Nuevo Leon, *Lophophora willliamsi* grows abundantly in the scrublands area, especially in Aramberri municipality. It is used to heal rheumatism and muscular pains, but, some residents told us (information recorded in the ethnobiological research, according to local people) that peyote and chaute *(Ariocarpus retusus*), another species abundantly found in this area, are also used by “hippies” as hallucinogenics. The plants are consumed in a crude form. Both, *Lophpophora williamsii* in north of Mexico and *Salvia divinorum* (called Shpherdess, leaves of Mary or Pipiltzintzintli) in the south, are hallucinogenic plants used for healing. Peyote is used crude or also crude and mixed with alcohol and marihuana, while Shepherdess leaves paste is smoked by people in Nuevo León (pers. com., out of the study area), Shepherdess has been reported as marihuan substitute [57].

### Similarities between ethnomedical studies in Mexico and other countries

Several species studied in southern Nuevo León have similar use in different societies worldwide such as *Citrus aurantifolia*, *Eucalyptus* spp. *Melissa oficinalis*, *Psidium guajava*, and *Carica papaya* studied in Mozambique [58]. These plants are used as medicinal species to heal cough, headache, high blood pressure, intestinal colic, and toothaches, respectively. The bark, roots, leaves, flowers and fruits of *Sambucus nigra, Thymus vulgaris* and *Olea europaea* are common medicinal plant used in the Iberian Peninsula [59]. All of these cultivated plants are present and used in the same way in southern Nuevo León. Of the 145 species of medicinal plants used to heal different diseases recorded in NW Argentina [60], some of them such as *Chenopodium ambrosioides, Erodium cicutarium, Rosmarinus officinalis*, and *Marrubium vulgare,* are also found in southern Nuevo León and are used in similar way. The most common uses of the 184 medicinal species recorded in Camagüey, Cuba were used to heal digestive disorders, skeletal muscle ailments, kidney problems and gynecological disorders, and the plant part most frequently used were leaves and fruit, prepared mainly by decoction and infusion [61]. Those species recorded and their usage are similar to those used in southern Nuevo León; Of the 92 plant species used in veterinary medicine recorded at Navarra Pyrinees [62], only four wild species, *Achillaea millefolium*, *Plantago major*, *Rorippa nasturtium-aquaticum*, and *Taraxacum officinale*, and 10 cultivated ones, *Allium cepa*, *A. sativum*, *Calendula officinalis*, *Marrubium vulgare*, *Melissa officinalis*, *Mentha spicata*, *Ocimum basilicum*, *Rosmarinus officinalis*, *Tanacetum parthenium*, and *Tymus vulgaris* reach their distribution in our study area, however, they are used in a different way.

## Conclusion

In southern Nuevo León people use at least 5% of the total State flora as medicinal plants, and most of these species are part of a few plant families. Most of medicinal species are wild and native. The two most important major plant communities, scrublands and oak-pine forest provide almost the same number of medicinal species. A third of the medicinal flora studied is used in all three municipalities, most of them are wild. Leaves, stems and fruits are the plant parts most commonly used for healing, and boiling is the most common method used. Men and women from the three municipalities know nearly the same number of species, but different number of uses. In two municipalities there was no correlation between the age and the number of species known. However, in Zaragoza, there was a high correlation between the age of those interviewed and the number of species known.

The heterogeneous and contrasting landscapes, climates, and vegetation types in the area have favored a rich plant diversity exploited by residents in different ways, allowing them to complement some of their needs in the short and medium term. The absence of modern industry forces residents to seek alternative sources of subsistence, in this way, the natural resources available are used for this purpose, and the utilization of plant species are crucial in this respect in their way of life.

## Competing interests

The authors declare that they have no competing interests.

## Authors’ contribution

The field work and database confection was carried out by EEC, BSM, MGL and JVQ, data analysis was carried out by EEC, BSM, JVQ, JJP, MPM, JSS, LSM and MCC. Manuscript preparation was conducted by all authors. All authors read and approved the final manuscript.

## Supplementary Material

Additional file 1 Appendix 1. **List of medicinal plants used in the southern region of the State of Nuevo León, México.** Number after plant author B.S. (Brianda Soto, number of collection), E.E (Eduardo Estrada, number of collection) Click here for file
