# Supplementary material for: Medicinal plants in the southern region of the State of Nuevo León, México
Source: J Ethnobiol Ethnomed. 2012 Dec 11;8:45. doi: 10.1186/1746-4269-8-45 (PMC3564779; doi:10.1186/1746-4269-8-45)
Supplement: Additional file 1 Appendix 1. — List of medicinal plants used in the southern region of the State of Nuevo León, México. Number after plant author B.S. (Brianda Soto, number of collection), E.E (Eduardo Estrada, number of collection) [file 1746-4269-8-45-S1.pdf]

Appendix 1. List of medicinal plants used in the southern region of the State of Nuevo León, México. Number after plant author B.S. (Brianda Soto, number of collection), E.E (Eduardo Estrada, number of collection)

Scientific name	Common name	Uses	Part used	Method of use
ADIANTACEAE				
Adiantum capillus-veneris L., B.S. 321	Culantrillo	Hemorrhages	Leaves and stems	Infusion
AGAVACEAE				
Agave americana L. var. *marginata* Trel., B.S. 416	Maguey	Beverage	Mead	Beverage
		Arthritis	(mead, aguamiel) Leaves cut into pieces embedded in alcohol	Poultice
Agave celsii Hook., B.S. 382	Maguey de peña	High fever and headache	Leaves	Roasted, stick over the temples
Agave lecheguilla Torr., B.S. 383	Lechuguilla	Baldness	Root	Macerated, boiled and mixed with shampoo
		Dandruff	Root	Macerated,

				boiled and mixed with shampoo
		Kidney stones	Root	Boiled, ingestion of infusion
		Prostate cancer	Root	Boiled, ingestion of infusion
Agave macroculmis Tod., B.S. 381	Maguey	Wounds	Mead (aguamiel) mixed with alcohol	Applied over the wound
		Beverage	Mead	Boiled, ingest
		Kidney stones	Mead	Boiled, ingestion of infusion, drink as the first meal of the day
		Diabetes	Sap	Spread on bread, ingest
		Food (quiote)	Scape (inflorescence)	Baked in a pit oven
		Encephalitis	Scape	Roasted, applied

		and wounds (animals)	(Inflorescence)	over the wounds
Aloe vera L., B.S. 415	Sábila	Diabetes and cholesterol	Leaves and pulp	Liquefied, together with *Opuntia ficus-indica*, ingest
		Kidney stone	Leaves and pulp	Liquefied, ingest
		Gastritis	Pulp	Leaves, peeled, boiled mixed with honey bee and wine, ingest; liquefied, together with piña and *Opuntia ficus-indica*, ingest
		Acne	Pulp	Mask on the face; with activated carbon for the skin spots
		Toothache	Leaves	Crude, applied in the affected

		area
Inflammation	Leaves	Roasted, applied as cataplasm
Blows	Leaves	Crude, applied as cataplasm
Varicose ulcers	Leaves	Crude, applied as cataplasm
Burns	Leaves	Crude, applied as cataplasm
Stomach inflammation	Leaves	Liquefied, together with *Opuntia engelmanii* and *Solanum tuberosum*, ingest
Flu	Leaves	Pulp, applied on the feet and throat
Abscesses	Leaves	Crude, chopped, eat every day
Backache	Leaves	Roasted, rub and massage with the pulp
Wounds	Leaves and pulp	Cataplasm,

				mixed with (or without) salt, as cataplasm
		Weight loss	Leaves	Liquefied, ingest
		Underarm odor	Leaves pulp	Spreads in the armpit
Sansevieria thyrsiflora Thunb., B.S. 322	Hierba de la víbora, guaco	Insect, spider and snakebite bites	Leaves	Pulp, mixed with *Allium sativum*, *Citrus limon* in alcohol; or boiled, applied in the affected area
Yucca carnerosana (Trel.) McKelvey, B.S. 379	Palma samandoca	Indigestion	Fruits	Roast, milled and boiled, together with *Artemisia ludoviciana*, ingest
Yucca filifera Chaubaud, B.S. 380	Palma china	Snakebite	Leaves	Pulp, applied as cataplasm
AMARANTHACEAE				
Amaranthus palmeri S. Watson, B.S. 414	Quelite	Constipation	Leaves	Salad, crude
ANACARDIACEAE				

Rhus virens Lindh. ex A. Gray, B.S. 378	Lantrisco	Diabetes	Bark	Boiled, ingestion of infusion
		Migraine	Resin	Spread on the temples
		Gums and loose teeth	Leaves	Chew, but not swallow
		High pressure	Leaves	Boiled, ingestion of infusion
Schinus molle L., B.S. 323	Pirul	Hives, rash and allergy	Leaves	Boiled, take a bath with infusion
		Loose teeth	Leaves	Mouthwash
		Cough	Leaves	Boiled, together with *Gnaphalium canescens* leaves and *Tamarindus indica* fruits, ingestion of infusion
		Kidney disorders	Leaves	Boiled, ingestion of infusion

		Menstrual colic	Fruits	Boiled, together with *Mentha piperita* and *Tagetes lucida* leaves, ingestion of infusion
		Headache	Leaves	Boiled, ingestion of infusion
		Fright	Whole plant	Pass it over the body
APIACEAE				
Apium graveolens L., B.S. 377	Apio	Cholesterol	Leaves and stems	Liquefied, together with *Citrus paradisi* and *Ananas comosus* fruit pulp, salad
		Diabetes	Leaves and stems	Liquefied, together with *Citrus paradisi*, ingest
Coriandrum sativum L., B.S. 413	Cilantro	Liver diseases	Leaves and stems	Boiled, ingestion of infusion

Cuminum cyminum L., B.S. 417	Comino	Itch and drool when babies are tetthing	Leaves and stems	Boiled with coconut fibers
Eryngium heterophyllum Engelm., B.S. 327	Hierba del sapo	Kidney and prostate diseases	Leaves	Boiled, ingestion of infusion
Foeniculum vulgare Mill., B.S. 412	Hinojo	Menstrual colic	Leaves and stems	Boiled, ingestion of infusion
		Abortive	Leaves and stems	Boiled, ingestion of infusion
		Stomach ache	Leaves	Boiled, ingestion of infusion
		Gastritis	Leaves	Boiled, ingestion of infusion
		Fright	Whole plant	Pass through the body
		Alcoholism	Leaves	Boiled, ingestion of infusion
ARACEAE				

Anthurium sp., B.S. 376	Lampazo	Rash and sunspost	Leaves	Applied over the affected area
ASTERACEAE				
Artemisia ludoviciana Nutt., B.S. 326	Estafiate o simonillo	Diarrhea	Leaves and stems	Boiled, ingestion of infusion
		Colic	Leaves and stems	Boiled, ingestion of infusion
		High fever	Leaves and stems	Boiled, ingestion of infusion
		Flu	Leaves and stems	Boiled, ingestion of infusion
		Promote appetite	Leaves and stems	Boiled, ingestion of infusion
		Indigestion	Leaves and stems	Boiled, ingestion of infusion
		Gastritis	Leaves	Boiled, together with *Mentha piperita*, ingestion of

				infusion
		Stomach ache and Constipation (adult)	Leaves	Boiled, together with *Marrubium vulgare*, ingestion of infusion
		Constipated (babies)	Leaves	Toasted, milled and boiled, together with *Matricaria recutita*, ingestion of infusion
Achillea millefolium L., B.S. 418	Real de oro	Colic, stomach ache and diarrhea	Leaves and stems	Boiled, ingestion of infusion
Ambrosia confertiflora DC., B.S. 324	Ambrosia	Stomach ache	Leaves	Boiled, ingestion of infusion
		Colic	Leaves	Boiled, together with *Marrubium vulgare* and *Mentha piperita* leaves and alcohol,

				ingestion of infusion
Baccharis salicifolia (Ruiz & Pav.) Pers., B.S. 436	Jarilla o escobilla	Fright	Whole plant	Pass it over the body
Calendula officinalis L., B.S. 419	Reynas	Headaches	Flowers	Boiled, ingestion of infusion
		Colic	Flowers	Boiled, ingestion of infusion
		Neuralgia	Flowers	Boiled, ingestion of infusion
		Cancer prevention	Flowers	Boiled, ingestion of infusion
		Tachycardia	Flowers	Boiled, together with *Litsea pringlei* and *Matricaria recutita* leaves, ingestion of infusion
		Chest pain	Flowers	Boiled, together with

				Pelargonium hortorum leaves, ingestion of infusion
Chrysactinia mexicana A. Gray, B.S. 325	Hierba de San Nicolás	Backache	Leaves and flowers	Boiled, ingestion of infusion
		Inflammation postpartum	Leaves and flowers	Boiled, together with *Mentha piperita* leaves, ingestion of infusion
		Menstrual cramps	Leaves and flowers	Boiled, ingestion of infusion
		Bronchitis	Leaves and flowers	Boiled, ingestion of infusion
		Stomach ache	Leaves and flowers	Boiled, ingestion of infusion
		Diarrhea	Leaves and flowers	Boiled, ingestion of infusion
		Headaches	Leaves and	Boiled, together

			flowers	with *Allium sativum* and *Allium cepa*, ingestion of infusion
		Sexual impotence	Leaves and flowers	Boiled, together with honey bee and *Coriandrum sativum* seeds, ingestion of infusion
		Infertility	Leaves and flowers	Boiled, ingestion of infusion for 27 days, non in menstruation, after 15 days, she can get pregnant
Dyssodia setifolia B. L. Rob., B.S. 411	Parraleña	Chills	Leaves	Boiled, ingestion of infusion
		Fever	Leaves	Boiled, ingestion of infusion

Flourensia cernua DC., B.S. 410	Hojasé	General pain	Leaves and stems	Boiled, ingestion of infusion
		Spleen	Leaves and stems	Boiled, ingestion of infusion
		Infections	Leaves and stems	Boiled, ingestion of infusion
		Diarrhea	Leaves and stems	Boiled, ingestion of infusion
		Stomach ache	Leaves	Boiled, ingestion of infusion or together with wine (optional), ingest
		Hindrance	Leaves	Toast and mill, mixing with oil and take a spoon, or the milled one boiled, and ingestion of

				infusion
Gochnatia hypoleuca (DC.) A. Gray, B.S. 375	Ocotillo	Cough	Leaves	Boiled, mixed with honey bee, ingestion of infusion
		Stomach ache	Leaves	Boiled, ingestion of infusion
		High pressure	Leaves	Boiled, ingestion of infusion
Gnaphalium canescens DC., B.S. 372	Gordolobo	Cough	Leaves and stems	Boiled, together with *Eucalyptus* and *Matricaria recutita* leaves; mixed with a tiny bit of *Pinus teocote* bark (inhaling vapors); with *Artemisia ludoviciana* leaves or with *Grindelia inuloides* leaves,

			honey bee and *Citrus limon* leaves, ingestion of infusion
	Expectorant	Whole plant	Boiled, ingestion of infusion
	Chest congestion	Whole plant	Boiled, ingestion of infusion
	Sinusitis	Leaves and stems	Boiled, ingestion of infusion
	Phlegm	Leaves and stems	Boiled, ingestion of infusion mixed with honey bee and lemon juice
	Sore throat	Whole plant	Boiled, ingestion of infusion
	Indigestion (babies)	Leaves and stems	Boiled, ingestion of infusion
	Stomach	Leaves and	Boiled,

		infection	stems	ingestion of infusion
Grindelia inuloides Willd. var. *inuloides*, B.S. 420	Árnica	Internal wounds	Leaves and flowers	Boiled, ingestion of infusion
		Gastritis	Leaves and flowers	Boiled, ingestion of infusion
		Inflammation	Leaves and flowers	Boiled, ingestion of infusion
		Diabetes	Leaves and flowers	Boiled, ingestion of infusion
		Hematoma	Leaves	Boiled, ingestion of infusion
		Pains	Leaves	Boiled, infusion applied as cataplasm
		Ulcer scar	Leaves	Boiled, ingestion of infusion
Gymnosperma glutinosum (Spreng.) Less., B.S. 421	Pegajosa	Broken bones	Leaves	Fractured leg is “plastered” with

		(animals)		leaves and covered with a bandage
Matricaria recutita L., B.S. 437	Manzanilla	Menstrual colic	Leaves, stems and flowers	Boiled, ingestion of infusion
		Inflammation postpartum	Leaves, stems and flowers	Boiled, ingestion of infusion
		High temperature	Leaves, stems and flowers	Boiled, ingestion of infusion
		Stomach ache	Leaves, stems and flowers	Boiled, ingestion of infusion, or (optional) together with *Aloysia triphylla*
		Eye infections	Leaves, stems and flowers	Boiled, solution applied with a cotton wad
		Hematoma	Leaves, stems and flowers	Boiled, solution applied to the wound with a cotton wad

Dizziness	Leaves, stems and flowers	Boiled, together with *Litsea pringlei* leaves and *Cinnamomum*, ingestion of infusion
Spleen	Leaves, stems and flowers	Boiled, together with *Litsea pringlei* leaves and/or *Citrus sinensis* fruit peel, ingestion of infusion
Measles, rubella and allergies	Leaves, stems and flowers	Boiled, take a bath with the infusion
Hair dye	Leaves, stems and flowers	Boiled, solution applied in the hair
Intestinal lavage	Leaves, stems and flowers	Boiled, ingest three tablespoons of flaxseed together with

				half onion, three times at day; boiled, together with flaxseed, *Flourensia cernua* leaves, and *Citrus lemon* juice ingestion of infusion,
		Grains (mouth)	Leaves, stems and flowers	Gargles with the solution
Tagetes lucida Cav., B.S. 422	Yerbaniz	Nervousness	Whole plant	Boiled, ingestion of infusion
		Insomnia	Whole plant	Boiled, ingestion of infusion
		Chills	Whole plant	Boiled, ingestion of infusion
		Bone pain	Whole plant	Boiled, ingestion of infusion
		Cough	Whole plant	Boiled,

			ingestion of infusion
	Internal pain	Whole plant	Boiled, together with canela and *Matricaria recutita*, ingestion of infusion
	Hair loss	Whole plant	Boiled, solution applied together with shampoo
	Snakebite	Whole plant	Dried plant into alcohol, wash the affected area
	Fright	Whole plant	Scrub the dried plant throughout the body
	Cramps	Whole plant	Dried plant into alcohol, wash throughout the affected area
	Leg fractures (cattle)	Leaves	Boiled, poultice with a gauze, the leg is “plastered” with

				leaves and covered with a bandage
Tanacetum parthenium Sch. Bip., B.S. 438	Altamisa	Stomachache	Leaves, stems and flowers	Boiled, ingestion of infusion
		Menstrual colic	Leaves	Boiled, ingestion of infusion, and rolled leaves soaked in alcohol place them in the stomach
		Diarrhea	Leaves, stems, and flowers	Boiled, ingestion of infusion
		Internal pain	Leaves	Boiled, ingestion of infusion
		Cough	Leaves	Boiled, ingestion of infusion
		Asthma	Leaves	Boiled, ingestion of

				infusion
		Earache and deafness	Leaves (meristem)	Rolled leaves, mixed with vick vaporub and put in the ear
		Fright	Whole plant	Scrub the dried plant throughout the body
Taraxacum officinale F.H. Wigg., B.S. 409	Diente de león	Liver	Whole plant	Boiled, ingestion of infusion
		Kidney infection	Whole plant	Boiled, ingestion of infusion
		Abortive	Whole plant	Boiled, ingestion of infusion
		Gumboil	Leaves	Boiled, infusion applied in affected area
		Hair loss	Leaves	Boiled, infusion together with *Jatropha dioica* and *Tragia ramosa* leaves,

				applied to the hair
		Promote menstruation	Whole plant	Boiled, ingestion of infusion
Trixis californica Kellogg var. *califórnica*, B.S. 374	Árnica	Internal and external wounds	Whole plant	Boiled, ingestion of infusion
		Gastritis	Whole plant	Boiled, ingestion of infusion
		Stomach ache	Whole plant	Boiled, ingestion of infusion
		Prostate cancer	Whole plant	Boiled, ingestion of infusion
		Indigestion	Whole plant	Boiled, ingestion of infusion
		Improve blood circulation	Whole plant	Boiled, ingestion of infusion
		Wash private parts	Whole plant	Boiled, infusion together with

				Aloe vera, apply three times at day
		Blows	Leaves and flowers	Infusion, apply to the affected area
		Reduce inflammation	Whole plant	Infusion, apply to the affected area
		Ulcer	Whole plant	Ingestion of infusion
		Mottled skin	Whole plant	Infusion, apply to the affected area
		Healing	Whole plant	Infusion, apply to the affected area
		Rheumatic pains	Whole plant	Boiled, let stand for several days together with “mezcal” and rub the affected area
Zinnia elegans Sessé & Moc., E.E. 22432	Cartulina	Cancer prevention	Flowers	Boiled, ingestion of

				infusion
BORAGINACEAE				
Borago officinalis L., B.S. 408	Borraja	Measles	Leaves	Boiled, ingestion of infusion
		Cough	Leaves	Boiled, together with canela and *Mentha piperita* leaves, ingestion of infusion
		High temperature	Leaves	Boiled, ingestion of infusion
		Infection caused by insects or spiders sting	Young leaves	Milled, spread on the affected part
Cordia boissieri A. DC., B.S. 407	Anacahuita	Cough	Bark, fruits and roots	Boiled, ingestion of infusion
Tiquilia canescens (DC.) A.T Richardson, B.S. 449	Ventosidad	Chest pain	Leaves	Boiled, ingestion of infusion
BRASSICACEAE				
Eruca sativa (L.) Mill., B.S.	Colesilla	Stomach	Whole plant	Boiled,

439		ache		ingestion of infusion
Lepidium virginicum L., B.S. 406	Hierba del pajarito	Stomach ache	Leaves and stems	Boiled, ingestion of infusion
		Diarrhea	Leaves and stems	Boiled, ingestion of infusion
		High fever	Leaves and stems	Boiled, ingestion of infusion
Rorippa nasturtium-aquaticum (L.) Schinz & Thell., B.S. 450	Berro	Cirrhosis	Leaves	Disinfected, eaten raw
Sisymbrium irio L, B.S. 451	Mostaza	Eye infection	Leaves	Boiled, solution applied into the eyes with a cotton wad
		Headaches	Seeds	Milled, apply (stick) on temples with *Pinus teocote* resin
		Earache	Leaves	Place a piece of leaf into the ear

		High temperature	Seeds	Milled seeds, apply (stick with fat) on the temples (“chiquiadores”)
		Mumps	Seeds	Milled, mixed with *Opuntia imbricata* pulp, place it in the affected area
BROMELIACEAE				
Tillandsia usenoides L. B.S. 466	Paistle	Burns	Leaves and flowers	Milled, mixed with glycerine, spread in affected area
BUDDLEJACEAE				
Buddleja scordioides Kunth., B.S. 405	Escobilla	Colic	Leaves and stems	Boiled, ingestion of infusion
		Stomach ache	Leaves	Boiled, together with *Marrubuim vulgare* or/and *Opuntia engelmannii* var. *cuija* root and

				Prosopis glandulosa bark, ingestion of infusion
Buddleja cordata Humb. ssp. *tomentella* (Standl.) E. M. Norman, B.S. 371	Tepozán	Wounds	Leaves	Boiled, infusion applied in the wound
		Toothache	Leaves	Smear fat on leaf, and adhered it in the affected area
		Neuralgia	Leaves	Boiled, ingestion of infusion
		Insects, spiders, and snakes bites	Leaves	Boiled, mixed with *Pinpinella anisum*, wash the wounds with the solution
		Anemia	Leaves	Boiled, ingestion of infusion
		Diabetes	Leaves	Boiled, ingestion of infusion

		Earache and eye pain	Leaves	Roasted, smear fat and attach it on the temples (“chiquiadores”)
		Hysterics	Leaves	Boiled, ingestion of infusion
		Bone pain	Leaves	Boiled, ingestion of infusion
CACTACEAE				
Ariocarpus retusus Scheidw., B.S. 401	Chaute	Calmative (Psychedelic)	Whole plant	Eaten crude
Cylindropuntia imbricata (Haw.) F.M. Knuth, B.S. 400	Coyonoxtle	Inflamed tonsils	Fruit	Roasted, macerated, applied externally on the throat
		Migraine	Fruit	Boiled, the first drink of the day before first meal
		Diabetes	Fruit	Eat raw
		Baldness	Fruit	Boiled, macerated, mixed with

				shampoo
		General wounds	Stems	Pulp applied as cataplasm
Cylindropuntia leptocaulis (DC.) F.M. Knuth, B.S. 370	Tasajillo	Dandruff	Fruits	Cut into pieces, put them in water for twelve hours, use as shampoo, the foam produced must be let act to eliminate dandruff
Echinocereus poselgeri Lem., B.S. 399	Sacasil	Broken bones	Root	Macerated, band the affected part, place the pulp over the band (not directly, the pulp is irritant to the skin if place directly) and band again, tight the bands, avoiding bones movement
		Burns	Root	Boiled, use as in

				broken bones
Lophophora williamsii (Salm-Dyck) J. M. Coult., B.S. 402	Peyote	Calmative (Psychedelic)	Whole plant	Eaten crude
Marginatocereus marginatus (DC.) Backeb., B.S. 404	Cardón u órgano	Fractured legs (animals)	Stems pulp	Macerated, applied as cataplasm and bandage it
Opuntia engelmannii Salm-Dyck ex Engelm. var. *cuija* Griffiths, B.S. 398	Nopal cuijo	Stomach ache	Stem pulp	Liquefied, mixed with pieces of mezquite bark, *Baccharis salicifolia* leaves, and few lemon drops, ingestion
Opuntia ficus-indica L., B.S. 403	Nopal de todo el año	Diabetes	Stem pulp	Liquefied together with *Aloe vera* pulp, ingest
		Tumors	Stem pulp	Cataplasm over the tumor
		Inflammation	Stem pulp	Cataplasm over the

				inflammation area
		High pressure	Stem pulp	Liquefied, ingest
		Constipation	Stem pulp	Liquefied, ingest
		Diarrhea and vomit	Root	Cut into pieces, boiled, ingest
		Gastritis	Stem pulp	Liquefied, ingest
CANNACEAE				
Canna indica L., B.S. 397	Coyol	Epidermical wounds	Leaves	Crude, smear fat and apply it as cataplasm over the wound
		Stomach ache	Leaves	Roasted, mixed with ambrosia leaves, pieces of onion, white and yolk of an egg, and yerbabuena, all of them embebed in alcohol, place the mix over the abdominal area,

				until pain disappear
		Pruritus	Root pulp	Macerated, applied directly over the grain(s)
		Inflammation	Root pulp	Macerated, applied directly over the inflammation area
CAPRIFOLIACEAE				
Sambucus mexicana Sarg., B.S. 396	Sauco	Bronchitis, cough	Flowers	Boiled, together with eucalypt leaves, honey bee, and *Bougainvillaea glabra* bracts, ingestion of infusion
		Asthma	Leaves	Boiled, ingestion of infusion
		High temperature	Leaves and flowers	Boiled, take a bath with the solution

		Inflammation	Flowers	Boiled, soak a towel, place over the inflammation
		Stomach ache	Flowers	Boiled, ingestion of infusion
		Pruritus	Bark	Macerated, together with sábila leaves and aspirin pill, the ointment resultant applied directly over the grain(s)
CHENOPODIACEAE				
Chenopodium ambrosioides L., B.S. 467	Epazote	Intestinal parasites	Leaves	Boiled in water or milk, mixed with *Mentha piperita* leaves, the first drink of the day before first meal
		Stomach ache	Leaves, stems and root	Boiled, ingestion of

				infusion
		Diarrhea	Leaves and stems	Boiled, ingestion of infusion
		Colic	Leaves	Boiled, ingestion of infusion
		Cough	Leaves and stems	Boiled, together with hierba del golpe leaves, ingestion of infusion
		Improve memory	Leaves and stems	Boiled, ingestion of infusion
Chenopodium graveolens Willd., B.S. 468	Epazote de zorrillo	Cough	Leaves and stems	Boiled, ingestion of infusion
		Bronchitis	Leaves and stems	Boiled, ingestion of infusion
		Kidney stones	Leaves and stems	Boiled, ingestion of infusion
		External	Leaves	Boiled, solution

		wounds		used to wash the wound
		Stomach ache	Leaves and stems	Boiled, ingestion of infusion
		Colic	Leaves and stems	Boiled, ingestion of infusion
		Suffocate	Leaves	Boiled, ingestion of infusion
		Constipate	Leaves	Boiled, ingestion of infusion
COMMELINACEAE				
Commelina dianthifolia [Delile,], B.S. 369	Hierba de la golondrina	Diarrhea	Leaves and stems	Boiled, ingestion of infusion
		Vomit	Leaves and stems	Boiled, ingestion of infusion
		High temperature	Leaves and stems	Boiled, ingestion of infusion
		Eyes	Flowers	Sap drops

		infection		applied directly over the eye; boiled in water and apply directly over the eye
		Children drools	Leaves and stems	Boiled, ingestion of infusion
		Inflammation	Leaves and stems	Boiled, together with *Trixis californica* var. *californica* leaves, wash the area and after that, apply honey bee
CONVOLVULACEAE				
Ipomoea purpurea (L.) Roth, B.S. 469	Correhuela	Insect bites	Leaves	Macerated, applied over the affected area
CRASSULACEAE				
Echeveria strictiflora A. Gray., B.S. 470	Siempreviva	Gumboil	Leaves	Macerated, mixed with edible oil and

				baking soda, apply over the gumboil
Sedum diffusum S. Watson, B.S. 395		Tachycardia	Leaves	Chew, macerate and apply it over the chest
CUCURBITACEAE				
Cucumis sativus L., B.S. 473	Pepino	Hair restoration	Fruit	Boiled, applied to the hair
Cucurbita foetidissima Kunth, B.S. 394	Calabacila loca	Hand cleaner	Fruit	Macerated, wash hands with the pulp
Cucurbita moschata Duchesne, B.S. 471	Calabaza	Backache	Fruit	Mashed, applied directly on the back
Sechium edule Sw., B.S. 472	Chayote	Improve blood circulation	Fruits	Cook, soup
CUPRESSACEAE				
Cupressus arizonica Greene, B.S. 393	Cedro	Spleen pain	Leaves and wood pieces	Boiled, together with canela, ingestion of infusion
Juniperus deppeana Steud.	Cedro	Vomit	Young branches	Boiled, ingestion of

				infusion
EQUISETACEAE				
Equisetum hyemale L. var. *affine* A. A. Eaton, B.S. 391	Cola de caballo	Kidney stones	Stems	Boiled together with *Zea mays* styles and *Eysenhardtia texana* wood pieces, ingestion of infusion
		Cancer prevention	Stems	Boiled, ingestion of infusion
ERICACEAE				
Arbutus xalapensis Sarg., B.S. 474	Madroño	Diabetes	Leaves	Boiled, ingestion of infusion
		Headache	Gum	Anoint on the temples
Arctostaphylos pungens Kunth, B.S. 392	Manzanita or pingüica	Kidney stones	Leaves	Boiled, together with *Equisetum hyemale* stems and *Zea mays* styles, ingestion of infusion

EUPHORBIACEAE				
Acalypha monostachya Cav., B.S. 476	Hierba del cancer	Cancer prevention	Leaves and stems	Boiled, ingestion of infusion
		Wounds and hematoma	Leaves and stems	Wash the wounded area; place the wound area over the steam produced by leaves and stems while boiling
Croton suaveolens Torr., B.S. 475	Salvia	Anemia	Stem pieces	Boiled, ingestion of infusion
		Diabetes	Stem pieces	Boiled, ingestion of infusion
		Low pressure	Leaves	Boiled, together with *Carya illinoiensis* bark, ingestion of infusion
		Headache	Stem pieces	Boiled, together with *Carya*

				illinoiensis bark, ingestion of infusion
		Fatigue	Leaves	Boiled, ingestion of infusion
		Amoeba	Leaves	Boiled, drink as the first drink of the day, and before the first meal of the day
Euphorbia antisyphilitica J. Meyrán, B.S. 478	Candelilla	Swollen feet	Root	Boiled, soak a towel and cover the foot
Euphorbia glyptosperma Engelm., B.S. 477	Hierba de la hormiga	Bladder infection	Whole plant	Boiled, ingestion of infusion
		Kidney infection	Root	Boiled, together with *Zae mays* styles and *Equisetum laevigatum* stems, ingestion of infusion
Jatropha dioica Sessé, B.S.	Sangre de	Warts	Gum	Applied directly

356	drago			over the wart
		Foot fungus	Root pieces	Boiled, introduce the feet into the solution
Tragia ramosa Torr., B.S. 457	Ortiguilla or mala mujer	General pain	Leaves	Boiled, ingestion of infusion
		Blood purification	Leaves	Boiled, ingestion of infusion
		Bronchitis	Leaves	Boiled, ingestion of infusion
		Evil spells	Whole plant	Boiled, ingestion of infusion
		Itch	Leaves	Boiled, ingestion of infusion
FAGACEAE				
Quercus mexicana Humb. & Bonpl., B.S. 459	Encino	Loose teeth	Bark	Milled, mixed with tooth paste, brush teeth
Quercus polymorpha	Encino	Cough	Fruit	Boiled,

Schltdl. & Cham., B.S. 460				ingestion of infusion
		Healthy teeth	Bark	Boiled, milled, mouthwash
GERANIACEAE				
Erodium cicutarium (L.) L´Hér., B.S. 461	Alfilerillo	Sore throat	Whole plant	Boiled, gargle
Pelargonium hortorum L. H. Bailey, B.S. 458	Geranio	Gingival inflammation	Leaves	Cataplasm, smeared with vegetable fat
		Wounds	Flowers and leaves	Milled, applied as cataplasm
		Toothache	Leaves	Chewing leaves
		Stomach ache	Leaves	Boiled, ingestion of infusion
		Headache	Leaves	Stick leaves with vick-vaporub in both temples
		Constipation (babies)	Part of petiole (oiled)	Introduce it into the anus
		Tachycardia	Flowers	Boiled, ingestion of infusion

		Hemorrhages	Flowers	Milled, applied as cataplasm in affected area
		Abortive	Leaves	Boiled, ingestion of infusion
JUGLANDACEAE				
Carya illinoniensis (Wangenh.) K. Koch, B.S. 424	Nogal	Hemorrhages	Fruit peel	Boiled, together with *Punica granatum* fruit peel, ingestion of infusion
		Diabetes	Bark	Boiled, ingestion of infusion
		Anemia	Bark and fruit peel together	Boiled, ingestion of infusion
		Blood purification	Leaves	Boiled, ingestion of infusion
		Cholesterol	Halves nut	Eaten raw
		Hair loss	Fruit peel	Add to shampoo
		Stomach inflammation	Halves nut	Blend with water, peanut

				and oatmeal
Carya myristiciformis (F. Michx.) Elliot, B.S. 479	Nogal cimarrón	Anemia	Leaves	Boiled, ingestion of infusion
LAMIACEAE				
Hedeoma drummondii Benth., B.S. 423	Poleo or regote	Sleeplessness	Leaves and stems	Boiled, ingestion of infusion
		Nervousness	Leaves	Boiled, ingestion of infusion
		Headache	Leaves	Place leaves on the temples
		Chills	Leaves	Crude milled leaves, inhale
		Cough	Leaves and stems	Boiled, ingestion of infusion
Hedeoma palmeri Hemsl., B.S. 480	Poleo de hoja ancha	Nervousness	Leaves	Boiled, ingestion of infusion
		Inflammation postpartum	Leaves	Boiled, ingestion of infusion
		Diabetes	Leaves and	Boiled,

			stems	ingestion of infusion
		Stomach inflammation	Leaves and stems	Boiled, ingestion of infusion
		Infertility	Flowers	Boiled, ingestion of infusion
		Menstrual colic	Flowers	Boiled, ingestion of infusion
		Insomnia	Leaves	Boiled, ingestion of infusion
Majorana hortensis Moench, B.S. 462	Mejorana	General aches	Leaves	Boiled, ingestion of infusion
		High temperature	Leaves	Boiled, ingestion of infusion
		Stomach ache	Leaves	Boiled, ingestion of infusion
		Menstrual colic	Leaves	Boiled together with

				Chenopodium ambrosiodes, ingestion of infusion
Marrubium vulgare L., B.S. 425	Marrubio	Hair loss prevention	Leaves and stems	Boiled and applied after shampoo
		Fungus foot	Leaves	Boiled, rinse foot on it
		Stomach ache	Leaves	Boiled, ingestion of infusion
		Diarrhea	Leaves and stems	Boiled, ingestion of infusion
		Indigestion	Leaves	Boiled, ingestion of infusion
		Hepatitis	Leaves and stems	Scrub the whole dry plant over the body and also ingestion of infusion
		Tinnitus	Apical meristem	Meristem into cotton roll and

				place it inside the ear
		Fright	Leaves and stems	Scrub the whole dry plant over the body
		Weight loss	Leaves	Boiled, the first drink of the day before first meal
		Spleen	Apical meristem	Boiled, ingestion of infusion
		Bad breath	Leaves	Boiled, mouthwash three times at day
		Wounds and grains	Leaves	Milled, applied as cataplasm
		Grinder ache	Leaves	Milled, applied as cataplasm, place in the affected area
		Smelly feet	Leaves	Place a bunch of leaves inside the shoes
Melissa officinalis L., B.S.	Toronjil	Tachycardia	Leaves	Boiled,

				ingestion of infusion
		Chest pain	Leaves	Boiled, together with *Ocimum basilicum* leaves, ingestion of infusion
		Calmative	Leaves	Boiled, ingestion of infusion
Mentha piperita L., B.S. 427	Yerbabuena or hierba buena	Stomach ache	Leaves	Boiled, ingestion of infusion
		Colic	Leaves	Boiled, ingestion of infusion
		Amoeba (babies)	Leaves	Boiled, ingestion of infusion
		High temperature	Leaves	Boiled, together with *Borago officinalis* leaves and canela, ingestion of infusion

	Flu	Leaves	Boiled, together with *Citrus limon* and *Borago officinalis* leaves, ingestion of infusion
	Cold	Leaves	Boiled, together with *Matricaria recutita* leaves and canela, ingestion of infusion
	Headache	Leaves	Boiled, ingestion of infusion
	Intestinal parasites	Leaves	Boiled, the first drink of the day before first meal
	External wounds	Leaves	Boiled, together with *Trixis californica* var. *claifornica* leaves, milled, applied in the

				affected area
		Indigestion	Leaves	Toasted, together with *Flourensia cernua* leaves and oil, take a spoonful
		Calmative	Leaves	Boiled, ingestion of infusion
		Vomit	Leaves	Boiled, ingestion of infusion
		Diarrhea	Leaves	Boiled, ingestion of infusion
Mentha spicata L., B.S. 368	Menta	Gastritis	Leaves and stems	Boiled, ingestion of infusion
		Insomina	Leaves and stems	Boiled, ingestion of infusion
Mentha rotundifolia Huds., B.S. 367	Mastranto	Stomach ache	Leaves and stems	Boiled, ingestion of infusion

		Indigestion (babies)	Leaves and stems	Boiled, ingestion of infusion
Monarda citriodora Cerv. var. *austromontana* (Epling) B. L. Turner, B.S. 384	Betónica cabezona	Diabetes	Leaves	Boiled, ingestion of infusion
		Menstrual delay	Leaves	Boiled, ingestion of infusion
Ocimum basilicum L., B.S. 328	Albahaca	Earache	Leaves	Mixed with olive oil, anoint it
		Envy	Whole plant	Scrub the whole dry plant over the body
		Nightmares	Whole plant	Place it at the bedside
		Chest pain	Whole plant	Toasted, inhaling vapors
		Bronchitis	Whole plant	Boiled, ingestion of infusion
		Vomit	Leaves or whole plant	Boiled or macerated crude, ingestion

				of infusion
		Fright	Whole plant	Scrub the whole dry plant over the body; boiled together with *Citrus sinensis* leaves, ingestion of infusion in the evenings
		Tachycardia	Leaves	Boiled, together with *Melissa officinalis* leaves, ingestion of infusion
		Stomach ache	Leaves	Boiled, ingestion of infusion
Poliomintha longiflora A. Gray, B.S. 329	Orégano	Cough	Leaves	Boiled, together with *Eucalyptus camaldulensis, Gnaphalium canescens* and *Cordia boissieri* leaves, ingestion of infusion;

				toasted and milled, mixed with fat, spreads on throat and feet
		Bronchitis	Leaves	Ingestion of infusion
Rosmarinus officinalis L., B.S. 385	Romero	Stained face	Whole plant	Clean the face with the infusion
		Insomnia	Leaves	Boiled, ingestion of infusion
		Stomach ache	Leaves	Boiled, ingestion of infusion; together with *Mentha piperita* leaves, ingestion of infusion
		High fever	Leaves	Boiled, together with *Majorana hortensis* and *Commelina dianthifolia* leaves, ingestion

			of infusion
	Hair loss	Whole plant	Boiled, use as shampoo
	Leg pain	Leaves	Boiled, rub with the infusion
	Fright	Whole plant	Scrub the whole dry plant over the body; boiled, together with *Leucophyllum frutescens* leaves, ingest and take a bath with the infusion
	Grey hair	Stem pieces	Embedded in alcohol, until turns green, applied in the hair
	Ward off witches	Whole plant	Nail a branch on the wall
	Gallblader	Whole plant	Boiled, together with *Achillaea millefolium* and canela, ingestion

				of infusion
Salvia chia Sessé & Moc., B.S. 426	Chia	Stomach ache	Stems and leaves	Boiled, ingestion of infusion
Teucrium cubense L., B.S. 448	Verbena	Blows	Leaves	Crude, as cataplasm mixed with egg white
		Kidney stones	Leaves and stems	Boiled, ingestion of infusion
		High temperature	Leaves	Boiled, ingestion of infusion, liquefied take a bath
		Fright	Leaves and stems	Scrub the whole dry plant over the body
		Stimultate apetite	Leaves	Boiled, ingestion of infusion
Thymus vulgaris L., B.S. 453	Tomillo	Diabetes prevention	Leaves	Boiled, ingestion of infusion
LAURACEAE				

Litsea glaucescens Kunth, B.S. 463	Laurel	Spleen	Leaves	Boiled, ingestion of infusion
		Chest pain	Leaves	Boiled, ingestion of infusion
Litsea pringlei Bartlett, B.S. 447	Laurel	Spleen	Leaves	Boiled, ingestion of infusion
		Headache	Leaves	Boiled, ingestion of infusion
Persea americana Mill. ssp. *drymifolia* (Schtldl. & Cham.) S. F. Blake, B.S. 428	Aguacate	Kidney	Seed	Boiled, together with *Equisetum hyemale*, *Zea mayz* and *Teucrium cubense* leaves, ingestion of infusion
		Amoebas	Leaves or seed	Boiled, ingestion of infusion
		Diarrhea	Leaves	Boiled, ingestion of

				infusion
		Gastritis	Leaves	Boiled, ingestion of infusion
		Hair growth	Seed and bark	Boiled, applied after shampoo
		Anemia	Leaves	Boiled, ingestion of infusion
		High pressure	Leaves and seed	Boiled, ingestion of infusion
		Cysts in matrix	Leaves	Boiled, ingestion of infusion
LEGUMINOSAE				
Acacia amentacea DC., B.S. 429	Chaparro prieto or gavial	Cancer prevention	Leaves and stems	Boiled, ingestion of infusion
Acacia farnesiana (L.) Willd., B.S. 332	Huizache	Kidney	Root and bark	Root boiled, together with *Opuntia engelmannii* var. *cuija* root, ingestion of

				infusion; bark boiled, together with *Prunus serotina* leaves, ingest in the morning and in the night
		Diarrhea	Stems	Boiled, together with *Celtis pallida*, *Buddleja scordioides*, *Chrysactinia mexicana* and *Lippia graveolens* leaves, ingestion of infusion
Eysenhardtia parvifolia Brandegee, B.S. 330	Vara dulce	Kidney stones	Stems	Boiled, ingestion of infusion
Eysenhardtia texana Scheele, B.S. 331	Vara dulce	Kidney stones	Stems and bark	Soak in water by one night, ingestion of solution

		Allergy	Stems	Boiled, take a bath with infusion
		Bladder infection	Bark	Soak in water by one night, ingestion of solution
Mimosa malacophylla A. Gray, B.S. 454	Charrasquilla	Kidney stones	Leaves and stems	Boiled, ingestion of infusion
Phaseolus vulgaris L., B.S. 464	Frijol	Diabetes	Seed	Cook, ingest
Prosopis glandulosa Torr. var. *torreyana* (L. Benson) M. C. Johnston, B.S. 446	Mezquite	Sore waist	Root	Macerated, together with *Opuntia* root, applied as cataplasm
		Incontinence (infants)	Resin (gum)	Smeared in the navel
		Alcoholism	Root	Boiled, ingestion of infusion (prevent relapse of alcoholism)
		Diarrhea	Leaves	Macerated,

			place in water for one o several days, ingestion of infusion
	Broken bones, fractures	Bark	Macerated, together with *Echinocereus poselgeri* root pulp, band the affected part, place the pulp over the band (not directly, the pulp is irritant to the skin if place directly) and band again, tight the bands, avoiding bones movement
	Stomach ache	Leaves	Boiled, together with *Matricaria recutita* leaves and *Buddleja scordioides* root,

				ingestion of infusion
		Indigestion	Wood hashes	Mixed with honey bee and ingest a spoon
Senna crotalarioides (Kunth) H. S. Irwin & Barneby, B.S. 333	Yerba del potro	Headache	Leaves	Boiled, ingestion of infusion
		Sore throat	Leaves	Milled, applied as cataplasm out of the throat
		Colic	Leaves	Boiled, ingestion of infusion
		Inflammation	Leaves	Boiled, ingestion of infusion
LILIACEAE				
Allium sativum L., B.S. 445	Ajo	Cough	Garlic clove	Mixed with *Citrus limon*; *Allium cepa*, *Citrus limon* juice and honey bee, ingestion of solution; boiled,

				together with canela, *Citrus limon* and honey bee, ingest
		Expectorant	Garlic clove	Crude, liquefied, the first drink of the day before first meal; toast and eat
		Constipation	Garlic clove	Boiled, ingestion of infusion
		Spider bite	Garlic clove	Crude, milled, applied as cataplasm in the affected area
		High pressure	Garlic clove	Boiled, ingestion of infusion
		High fever	Garlic clove	Boiled, ingestion of infusion
		Diabetes	Garlic clove	Ingest crude
		Typhoid	Garlic clove	Ingest crude
Asphodelus fistulosus L.,	Cebollín	Stained face	Stems	Macerated,

B.S. 334				applied as cataplasm, clean the face
LORANTHACEAE				
Phoradendron villosum (Nutt.) Nutt. ex Engelm., B.S. 430	Injerto	Cancer prevention	Leaves	Boiled, ingestion of infusion
		Kidney stones	Leaves and stems	Boiled, ingestion of infusion
		Diabetes	Leaves and stems	Boiled, ingestion of infusion, ingest in the morning and in the night
		Grinder ache	Leaves	Chewing leaves
		Urinary problems (horses)	Leaves and stems	Place leaves and stems together with four liters of water, boil it until vaporize half of the content, give it to drink the horse

		Anemia	Leaves	Boiled, ingestion of infusion
LYTHRACEAE				
Heimia salicifolia Link & Otto, B.S. 431	Jara	Eye infections	Leaves	Boiled, together with *Fraxinus cuspidata*, infusion applied into the eyes
MALVACEAE				
Malva parviflora L., B.S. 335	Malva	Abscesses	Leaves	Macerated crude, applied as cataplasm
		Varicose ulcers	Leaves	Macerated crude, applied as cataplasm
		Grinder ache	Leaves	Chewing leaves
		Burns	Leaves	Macerated crude, applied as cataplasm together with oil
		Blows	Leaves	Macerated crude, applied as cataplasm
		Swollen feet	Leaves	Macerated

		crude, applied as cataplasm
Wounds	Leaves	Boiled, wash affected part with the infusion
Grains	Leaves	Macerated crude, applied as cataplasm
High temperature	Leaves and stems	Boiled in two liters of water, with the infusion take a bath
Sore hands	Leaves	Boiled, with the infusion wash the hands
Nervousness	Leaves	Boiled, together with *Plantago major*, ingestion of infusion
Stomach ache	Leaves	Boiled, ingestion of infusion
Tinnitus	Leaves	Crude, stick leaves with fat on the ear

		Throat infection	Leaves	Boiled, infusion applied out of the throat
MORACEAE				
Ficus carica L., B.S. 336	Higo	Nosebleed	Leaves	Toasted, inhaling the smoke
		Wounds	Fruit	Pulp, applied directly over the wound
		Pustule	Fruit pulp or/and sap	Applied directly to the wound
Morus celtidifolia Kunth, B.S. 455	Mora	Blows	Leaves and stems	Milled, applied as cataplasm
MYRTACEAE				
Eucalyptus camaldulensis Dehnh., B.S. 337	Eucalipto	Cough	Leaves	Boiled, together with *Gnaphalium canescens* leaves, ingestion of infusion
		Tired muscles	Leaves	Milled, together with glycerin, *Ruta graveolens* leaves, onion

				and garlic pieces, alcanfor and alcohol, rub the muscles
Psidium guajava L., B.S. 444	Guayaba	Diarrhea	Fruit	Eat crude
		High pressure	Fruit	Liquefied, drink
NYCTAGINACEAE				
Bougainvillea glabra Choisy, B.S. 456	Bugambilia	Cough	Bracts and flowers	Boiled, together with *Eucalyptus camaldulensis* and *Gnaphalium canescens* leaves, ingestion of infusion
OLEACEAE				
Fraxinus cuspidata Torr., B.S. 465	Fresno	Back pain	Branches	Lying on them for four consecutive nights
Olea europea L., B.S, 443	Olivo	Improve blood circulation and	Leaves	Boiled, ingestion of infusion

		cholesterol		
ONAGRACEAE				
Oenothera rosea Aiton, B.S. 338	Hierba del golpe	Blows, external wounds	Leaves	Milled, mixed with fat, apply as cataplasm
PINACEAE				
Pinus cembroides Zucc., B.S. 432	Piñonero	Rheumatism	Resin	Melted, applied as cream
		Broken bones (animals)	Resin	Fractured leg is "plastered" with resin, together with *Opuntia imbricata* stem pulp, and covered with a bandage
		Bronchitis	Young branches	Boiled, together with brown sugar, ingestion of infusion
		Leg pain	Resin	Melted, together with alcohol, applied as cataplasm
		Back injuries	Resin	Melted, applied

		(donkeys)		to the wound
Pinus pseudostrobus Lindl. var. *estevezii* Martínez, B.S. 339	Pino blanco	Kidney disorders	Bark	Boiled and milled, together with *Prunus serotina* milled bark, ingestion of infusion
PLUMBAGINACEAE				
Plumbago pulchella Boiss., B.S. 442	Júrica or Júdica	Swollen waist	Leaves	Boiled, wash the affected part
POACEAE				
Avena fatua L. var. *sativa* Thell., B.S. 441	Avena	Cholesterol	Seeds	Boiled, together with almonds, ingest
		Allergies	Seeds	Boiled, take a bath
Cymbopogon citratus Stapf., B.S. 340	Zacate limon	Flu	Leaves	Boiled, ingestion of infusion
		Nervousness	Leaves	Boiled, ingestion of infusion
		Intestinal parasites	Leaves	Boiled, ingestion of infusion

		Inflammation	Leaves	Boiled, together with nutmeg and *Croton suaveolens* leaves, ingestion of infusion
Phalaris canariensis L., B.S. 342	Alpiste	High pressure	Leaves and stems	Boiled, together with *Hedeoma palmeri* leaves, ingestion of infusion
		Improve blood circulation	Leaves and stems	Boiled, soak in two liters of water, ingestion of infusion daily
Triticum aestivum L., B.S. 341	Trigo	Cholesterol	Seeds	Liquefied, together with almonds and walnuts, ingest
		Ingrown toenalis	Seeds	Chew, applying to the affected part covering with a bandage for few days
Zea mays L. ssp. *Mays*, B.S.	Maíz	Kidney	Styles (female	Boiled, together

433		disorders	flowers)	with *Verbena carolina* leaves, lemon juice and potato peel, ingestion of infusion
		Kidney stones	Styles (female flowers)	Boiled, together with *Arctostaphylos pungens* leaves and *Equisetum laevigatum* stems, ingestion of infusion
PORTULACACEAE				
Portulaca mundula I. M. Johnst., B.S. 366	Verdolaga	Indigestion	Leaves	Boiled, ingestion of infusion
		Constipation	Leaves	Boiled, ingestion of infusion
		Uric acid	Leaves	Boiled, ingestion of infusion
PUNICACEAE				

Punica granatum L., B.S. 343	Granada	Hemorrhage	Fruit	Boiled, ingestion of infusion
		Broken tooth	Fruit peel	Boiled, as mouthwash
RANUNCULACEAE				
Clematis drummondii Torr. & A. Gray, B.S. 434	Barba de chivo	Expectorant	Whole plant	Macerated and inhalation
		Skin infection	Whole plant	Boiled, take a bath
RHAMNACEAE				
Karwinskia humboldtiana Zucc., B.S. 440	Coyotillo or tullidora	Brucellosis (goats)	Leaves	Boiled, ingestion of infusion
ROSACEAE				
Crataegus tracyi Ashe var. *madrensis* J. B. Phipps, B.S. 344	Tejocote	Kidney disorders	Root	Macerated and boiled, ingestion of infusion
		Clots	Root	Macerated and boiled, ingestion of infusion
		Bowel inflammation	Fruits	Boiled, ingestion of infusion

		Weight loss	Fruits	Boiled, ingestion of infusion
		Hemorrhage	Fruits	Boiled, together with *Aloe vera* and *Matricaria recutita* leaves, ingestion of infusion
		Anemia	Fruits	Boiled, ingestion of infusion
		Diabetes	Root	Macerated and boiled, ingestion of infusion
Eriobotrya japonica (Thunb.) Lindl., B.S. 345	Níspero	High pressure	Leaves	Boiled, ingestion of infusion
		Diabetes	Fruits	Boiled, ingestion of infusion
		Diarrhea	Fruits	Eat crude
Prunus domestica L., B.S. 435	Ciruelo	Constipation and indigestion	Fruit	Eat crude

Prunus persica (L.) Batsch, B.S. 346	Durazno	Amoeba	Leaves and fruits	Boiled, ingestion of infusion
		Intestinal parasites	Leaves and root	Boiled, ingestion of infusion
Prunus serotina Ehrh. ssp. *capuli* (Cav. ex Spreng.) McVaugh, B.S. 354		Nervousness	Leaves	Boiled, ingestion of infusion
		Kidney disorder	Bark	Boiled, together with *Pinus pseudostrobus* milled bark, ingestion of infusion
		Bladder infection	Bark	Boiled, together with *Trixis californica* var. *californicq, Equisetum laevigatum, Mimosa biuncifera, Borago*

				officinalis, and *Buddleja cordata* leaves, ingestion of infusion
Purshia plicata (D. Don) Henrickson, B.S. 353	Rosa de castilla de monte	Diarrhea	Leaves and flowers	Boiled, ingestion of infusion
		High fever	Leaves and flowers	Milled, mixed with fat, rub the body
		Stomach ache	Leaves and flowers	Boiled, ingestion of infusion
		Diabetes (prevention)	Leaves and flowers	Boiled, ingestion of infusion, drink it regularly
		Constipation	Leaves and flowers	Boiled, ingestion of infusion
Rosa gallica L., B.S. 352	Rosal	High temperature	Leaves, stems and fruits	Boiled, ingestion of infusion
Rubus flagellaris Willd.,	Zarzaparrilla	Anemia	Leaves and	Boiled,

B.S. 365			stems	ingestion of infusion
		Diabetes	Leaves and stems	Boiled, ingestion of infusion
		Kidney disorders	Leaves and stems	Boiled, ingestion of infusion
RUTACEAE				
Citrus limon (L.) Burm., B.S. 351	Limón	Nervousess	Leaves	Boiled, together with *Citrus sinensis* leaves, ingestion of infusion
		Cough and asthma	Fruit juice	Ten lemons, mixed with one onion and three garlic cloves (liquefied), ingest it
Citrus sinensis Osbeck, B.S. 347	Naranja	Nervousness	Leaves; flowers	Boiled, together with *Citrus sinensis* leaves, ingestion of infusion; boiled,

			ingestion of infusion
	Insomnia	Leaves	Boiled, ingestion of infusion
	Sore knees	Leaves	Boiled, ingestion of infusion
	Uric acid	Leaves	Boiled, ingestion of infusion
	Uterus cysts	Flowers	Boiled, together with nutshell, avocadobone pieces, *Rosmarinus officinalis, Pimpinella anisum, Trixis californica,* and *Chrysactinia mexicana* leaves, ingestion of infusion

		Internal hemorrhages	Leaves	Boiled, together with *Punica granatum* peel, ingestion of infusion
		Headache	Leaves	Boiled, ingestion of infusion
Ruta graveolens L., B.S. 348	Ruda	Toothache	Leaves and flowers	Boiled, ingestion of infusion
		High pressure	Leaves	Boiled, ingestion of infusion
		Insomnia	Leaves	Boiled, ingestion of infusion
		Nervousness	Leaves and flowers	Boiled, ingestion of infusion
		Allergies	Leaves	Boiled, take a bath
		Hangover	Leaves	Boiled, ingestion of infusion

Ringing in the ears	Leaves	Boiled, mixed with alcohol, applied in the ears
Stomach ache	Leaves	Boiled, ingestion of infusion
Diarrhea	Leaves	Boiled, ingestion of infusion
Abortive	Leaves and flowers	Boiled, ingestion of infusion
Colic	Leaves	Boiled, ingestion of infusion
Rheumatism	Whole plant	Boiled, mixed in alcohol, rub the affected part
Ward off witches	Whole plant	Place a ruda cross at bedside
Stomach ache	Leaves	Boiled, ingestion of infusion
Migraine	Leaves	Boiled,

				ingestion of infusion
		Deafness	Leaves	Crude, introduce young leaves on the ears
SALICACEAE				
Salix lasiolepis Benth., B.S. 355	Taray	Diabetes	Leaves	Boiled, ingestion of infusion
SCROPHULARIACEAE				
Leucophyllum frutescens (Berland.) I. M. Johnst., B.S. 364	Cenizo	Hepatitis	Whole plant	Boiled, ingestion of infusion, and take a bath with it
Maurandya antirrhiniflora Humb. & Bonpl. ex Willd., B.S. 373	Juan y mipil	Measles	Whole plant	Milled, applied on the skin
SELAGINELLACEAE				
Selaginella pilifera A. Br., B.S. 349	Flor de peña	Gastritis	Leaves	Boiled, ingestion of infusion
		Kidney disorders	Leaves	Boiled, ingestion of infusion

SIMAROUBACEAE				
Castela erecta Turp. var. *texana* (Torr. & A. Gray), Cronquist, B.S. 350	Bisbirinda	Amoeba	Leaves	Boiled, ingestion of infusion
		Constipation	Leaves	Boiled, ingestion of infusion
		Pancreatitis	Leaves	Boiled, ingestion of infusion
		Stomach ache	Leaves	Boiled, ingestion of infusion
SOLANACEAE				
Capsicum annuum (Dunal) var. *glabriusculum* Heiser & Pickersgill, B.S. 356	Chile de pájaro	Diarrhea	Leaves and stems	Boiled, ingestion of infusion
Capsicum annuum L. var. *annum,* B.S. 386	Chile piquín	Diarrhea	Leaves and stems	Boiled, ingestion of infusion
Nicotiana glauca Graham, B.S. 363	Gigante	Knee pain	Leaves	Macerated, toasted, mix with fat, rub the affected part
Physalis philadelphica	Tomatillo	Cough	Calyx	Boiled,

Lam., B.S. 362				ingestion of infusion
Solanum tuberosum L., B.S. 357	Papa	Gastritis	Root	Liquefied, ingestion crude
		Inflamed eyes	Root	Slices, put on the eyes
		Colitis	Root	Liquefied, together with lemon juice, the first drink of the day before first meal, for ten consecutive days
TRAPEOLACEAE				
Tropaeolum majus L., B.S. 361	Mastuerzo	Headache	Leaves	Stick over the temple
ULMACEAE				
Celtis laevigata Willd., B.S. 387	Palo blanco	Diabetes	Bark	Boiled, ingestion of infusion
		Kidney disorders	Bark	Boiled, ingestion of infusion
		Grains	Leaves and bark	Boiled, wash the affected part

Celtis pallida Torr., B.S. 390	Granjeno	Ingrown toenails	Leaves and fruits	Milled, mixed with fat, applying the affected part, and covered with a bandage
VERBENACEAE				
Aloysia triphylla Britton, B.S. 360	Cedrón	Stomach ache	Leaves	Boiled, together with *Matricaria recutita* leaves, ingestion of infusion
		Menstrual cramps	Leaves	Boiled, ingestion of infusion
		High pressure	Leaves	Boiled, ingestion of infusion
Glandularia bipinnatifida Nutt., B.S. 359.	Moradilla	The evil eye	Whole plant	Milled, into cold water, rub the whole body with the solution
Lippia graveolens Kunth., B.S. 388	Pionia	Headache	Leaves	Boiled, ingestion of infusion

VITACEAE				
Vitis cinerea (Engelm.) Millardet, B.S. 358	Uva silvestre	Backache	Stems	Boiled, ingestion of infusion, and rub the back with the solution
ZYGOPHYLLACEAE				
Larrea tridentata Coville, B.S. 389	Gobernadora	Stomach ache	Stems	Boiled (for 10 seconds, it is very strong plant), ingestion of infusion or chew the meristems for few seconds (not swallow) and spit out
		Diarrhea	Leaves	Boiled, ingestion of infusion
		Kidney disorders	Leaves and stems	Boiled, ingestion of infusion
		Foot pain	Leaves and stems	Boiled, rub the affected part

		with the solution
Constipation	Stems	Boiled (for 10 seconds, it is very strong plant), together with *Flourensia cernua* leaves, ingestion of infusion
Foot fungus	Whole plant	Boiled, rub the foot with the solution for 30 minutes
Kidney stones	Whole plant	Boiled (for 10 seconds, it is very strong plant), ingestion of infusion
Gastritis	Leaves	Place the leaves in water for one night, ingestion of infusion
Smelly feet	Leaves	Put a bunch of leaves into shoes or boil them,

			together with salt and wash the feet
	Fright	Whole plant	Pass it over the body
